# Salmagundi

**Published:** 1887-07

**Authors:** 


### SALMAGUNDI.

EDITED BY E. G. BETTY, D.D.S.

PLASTER OF PARIS BANDAGES.—To remove plaster of Paris bandages, paint a strip across at the desired point of division with a camel's hair pencil, dipped into nitric acid or vinegar. The acid will soften the plaster so that it may readily be cut with a knife.

TANNIN FOR BURNS.—An ethereal solution of tannin, of syrupy consistence, is said to be the best application to burns. It immediately soothes the intense pain, dries rapidly, and forms a pliable, non-elastic coating which is preferable to colodion, because it does not shrink and become stiff.

FREEZING.—Dr. Ward says: A few days ago the tips of three of the fingers of my right hand were frozen. They were stiff and felt as if burned by fire—for a frost bite is a burn. I applied strong spirits of ammonia to them. Three applications made a complete cure, removing all stiffness and soreness.

COUGH.—A strong and sonorous cough suggests spasmodic croup. A hoarse and rough cough is an indication of true croup. When the cough is clear and distinct there is bronchitis. When it is suppressed and painful there is pneumonia and pleurisy. If the cough is convulsive it indicates whooping cough.

EULYPTOL.—This is a mixture of salicylic acid, six parts; carbolic acid, one part; and oil of eucalyptus, one part, which Dr. Schmeltz believes to be preferable to most other antiseptics. It has a strong aromatic odor, and an acrid, burning taste; dissolves readily in alcohol, ether, chloroform, and a mixture of equal parts of alcohol and glycerine—also in alkalies, but is sparingly soluable in water. Urine to which a small quantity of eulyptol has been added remains unchanged for fully a month.

STICKY FLY-PAPER.—Take heavy unsized paper (Manilla preferable), paint with common glue and let dry; then spread the paper with the following mixture:

Castor oil....................................2 oz.
Resin...........................................6 oz.
Sugar...........................................1 oz.

Melt over a slow fire, stirring constantly; spread while yet warm.—*Formulary.*

---

An Anodyne for Dental Caries.—The following mixture is recommended:

R. Camphor.........................................5 parts
Chloral hydrate...............................5 parts
Cocaine hydrochloride......................1 part

On heating this mixture to the boiling point of water, an oily liquid is formed. This is to be applied lightly, and is said that after a few applications the pain will surely be alleviated.

---

Mr. Geo. J. Romanes has communicated to the Linnean Society the results of some experiments made by him to test the sense of smell in dogs. He finds that not only the feet, but the whole body of a man exhales a peculiar odor which a dog can recognize as that of his master amidst a crowd of other persons; that the individual quality of this odor can be recognized at great distances to windward, or in calm weather at great distances in any direction; and that even powerful perfumes may not overcome this odor. Yet a single sheet of brown paper, when stepped upon instead of the ground, and afterward removed, was sufficient to prevent the dog from following his trail.

---

Some of the features of short-hand-writing, synchronous-multiplex telegraphy, and type-writing, are combined in a system of steno-telegraphy invented by M. G. A. Cassagnes of Paris. In recent experiments over a wire running from Paris to Orleans and back, messages were sent at the rate of two hundred words a minute, that being the highest speed attainable by a nimble-fingered operator. By means of an automatic transmitting apparatus, using a strip of paper previously perforated, as in some of the systems of telegraphy already in vogue, seventeen thousand words per hour were sent over a line 650 kilometres in length, the messages being automatically printed by the receiving instrument.

THE decade between twenty and thirty is found to be the critical one in the formation of intellectual and professional habits, while the period of life before twenty is the most important for the fixing of personal habits. From this it follows easily that by education we must seek " to make automatic and habitual, as early as possible, as many useful actions as we can," and, conversely, to prevent the dropping into injurious habits. Professor James shows how unconsciously habits of mind are formed through the process of our daily routine, until some day we awake to the fact that we have acquired peculiar power or skill in some direction. The constant preaching of this truth would infuse new hope and ambition into many desponding workers.

